# Chemical Investigations in *Kelussia odoratissima* Mozaff. Leaves Based on Comprehensive Analytical Methods: LC-MS, SPME, and GC-MS Analyses

**DOI:** 10.3390/molecules28166140

**Published:** 2023-08-19

**Authors:** Mehdi Rahimmalek, Antoni Szumny, Shima Gharibi, Natalia Pachura, Mehran Miroliaei, Jacek Łyczko

**Affiliations:** 1 Department of Food Chemistry and Biocatalysis, Wrocław University of Environmental and Life Sciences, 50-375 Wrocław, Poland; natalia.pachura@upwr.edu.pl (N.P.); jacek.lyczko@upwr.edu.pl (J.Ł.); 2 Department of Horticulture, College of Agriculture, Isfahan University of Technology, Isfahan 84156-83111, Iran; 3 Core Research Facilities (CRF), Isfahan University of Medical Sciences, Isfahan 81746-73461, Iran; s.gharibi@mail.mui.ac.ir; 4 Faculty of Biological Science and Technology, Department of Cell and Molecular Biology and Microbiology, University of Isfahan, Isfahan 81746-73441, Iran; miroliaeimehran@gmail.com

**Keywords:** *Kelussia*, LC-MS, phenolic, flavonoid, oil, GC-MS, SPME

## Abstract

*Kelussia odoratissima* Mozaff. is a species of Apiaceae endemic to the Zagros Mountains in Iran. In the present investigation, for the first time, the polyphenolic compounds and flavonoids of its leaves were determined by liquid chromatography-mass spectrometry (LC-MS). As a result, *p*-coumaric acid, ferulic acid, caffeic acid, chlorogenic acid, acetyl phloroglucinol, vanillic acid, *m*-coumaric acid, and 4-methylsiringol were determined as the main phenolic compounds, while 3-hydroxyflavone, flavone, quercetin, rutin, neohesperidin, polydatin, and diosmin were the main flavonoid components, of which chlorogenic acid (303.08 µL/gDW), neohesperidin (38.37 µL/gDw), and diosmin (28.62 µL/gDW) were the most abundant. Solid-phase microextraction (SPME) was also used to determine the chemical compounds. Based on SPME, (*Z*)-undec-6-en-2-one (17.48%) and (*Z*)-butylidenephthalide (4.348%) were the major components. Based on GC-MS analyses, (*Z*)-ligustilide was the main compound; however, some new compounds were also determined, including 3-ethylisobenzofuran-1 (3H)-one, (*E*)-ligugustilide, and *E*-*n*-butylidene phthalide. Also, for the first time, we have identified EOs ethyl and isobutyl phthalides on the basis of the obtained EI-MS spectra. Finally, the fragmentation of phthalides is also discussed in this research.

## 1. Introduction

*Kelussia odoratissima* Mozaff. belongs to the Apiaceae family and is an endemic plant of Iran. It grows mainly in the western part of the Zagros Mountains. This species is an aromatic medicinal plant that is mostly used as a spice as well as for some therapeutic purposes [1]. It is an endangered species because of rough harvesting of the plants in natural habitats. *Kelussia* dried herbs have been consumed as traditional spices by the local people. In addition, both the leaves and seeds have been used for food and pharmaceutical purposes [2,3]. For instance, the fresh parts have been used in preparation prickles products. For *Kelussia* herbs, different activities have been reported such as sedative and anxiolytic [4], cytotoxicity [5], antioxidant [6], analgesic, and anti-inflammatory activities [7].

The application of different analytical methods under one experimental condition and the same plant material can provide new information for further decision making about the studied plant’s use in different food or pharmaceutical industries. From this point of view, volatiles and non-volatiles are considered crucial components of the plant for further industry processes. Gas chromatography based on mass spectrophotometry (GC-MS) is used for determining the terpenoids, while the solid-phase microextraction (SPME) method is a valuable method for determining the aroma of the plants. Finally, liquid-chromatography-mass spectrometry (LC-MS) is a robust method for non-volatile identifications [8]. Since SPME data mostly apply to the determination of the aroma of plants, it can provide more detailed information regarding the aroma in spice plants like *Kelussia*. Furthermore, the combination of SPME and oil GC-MS analyses can provide new insights for further sensory analyses for use of the spice in some food products.

Most previous reports on the determination of *Kelussia* focused on essential oil components in different habitats [3], different organs [9], and different drying methods [10]. As predominated compounds such as alkyl-alkenyl phthalide derivatives were detected in *Kelussia* leaves, e.g., isomers of ligustilide, and *cis*-3-butyldene phthalide [10], they were affected by drying treatments but no isobutyl- or ethyl phthalides were detected in this report. Alkyl or alkenyl phthalides are found in plants from the Apiaceae family, e.g., *Levisticum*, *Apium*, *Angelica*, *Cnidium*, *Ligusticum* [11]. Moreover, there are previous studies on the composition of oil in *Kelussia* is reported; however, there are no reports regards the fragmentation of phthalides in this species. 

As many health benefit compounds are categorized in the non-volatile group of compounds, determination of these components can be further beneficial for formulations of pharmaceutical or food products. However, there is no report regarding the determination of non-volatiles, especially phenolic and flavonoid compounds of this endemic species. Moreover, this is the first comprehensive report to use three different analytical methods for the determination of volatiles and non-volatiles in *K. odoratissima*.

The objective of this study is to determine the polyphenolic compounds of *K. odoratissima* based on LC-MS, as well as the oil components, and the aroma using GC-MS and SPME analyses.

## 2. Results and Discussion

### 2.1. Phenolic and Flavonoid Compounds

In the present research, phenolic and flavonoid compounds were determined using LC-MS analysis. Table 1 illustrates the details of the MRM mode analysis for the identified compounds. Accordingly, *p*-coumaric acid, ferulic acid, caffeic acid, chlorogenic acid, acetyl phloroglucinol, vanillic acid, *m*-coumaric acid, and 4-methylsiringol were determined as the main phenolic compounds. Among the phenolic acids, chlorogenic acid (303.08 µL/gDw of the sample) and 4-methylsiringol (57.03 µL/gDw) were revealed to be present in the highest amounts, while caffeic acid (1.94 µL/gDw) showed the lowest value (Table 2). In previous research, among polyphenolics, only ferulic acid was determined in *Kelussia* aerial parts based on vacuum- LC techniques [12]

Different types of flavonoids were also observed in the *Kelussia* sample. Based on LC-MS data, 3-hydroxyflavone, flavone, quercetin, rutin, neohesperidin, polydatin, and diosmin were the major flavonoid components in the *Kelussia* leaves. Consequently, neohesperidin (38.4 ± 0.87 µg/gDw) and diosmin (28.62 ± 0.76 µg/gDw) were the main flavonoid compounds (Table 3). Interestingly, neohesperidin was established for the first time in the leaves of *Kelussia*. However, hesperidin and diosmin were also reported in *Crithmum maritimum*, a halophyte plant of the Apiaceae family [13]. Diosmin was also reported in the seeds of *Notopterygium franchetii* from the Apiaceae family [14]. These flavonoids possess similar structures and activities such as hypolipidemic, diuretic, anticancer, and anti-hypertensive qualities [13]. Moreover, previous studies have revealed that diosmin can prevent fat accumulation and glucose intolerance with high anti-dyslipidemic effect [14]. Furthermore, Moieni et al. [15] also reported the anti-diabetic and anti-atherosclerotic properties of *Kelussia* which might be attributed to some groups of flavonoids like diosmin. Based on previous research, the flavonoid patterns of fruits and leaves are different, as Khanavi et al. [1] determined five flavonol glycosides including isorhamnetin 3-O-glucoside, quercetin 3-O-glucoside, isorhamnetin 3-O-rutinoside, isorhamnetin 3-O-glucuronide, and quercetin 3-O-glucuronide in *Kelussia* fruits. Finally, based on the literature, the polyphenolic and flavonoid pattern of *Kelussia* leaves is more similar to *Ferula orientalis* L. since in this species chlorogenic acid and diosmin were the main polyphenolic compounds [16].

### 2.2. GC-MS SPME-Arrow Results

Table 4 illustrates the results of SPME. For this analysis, according to the SPME data, 50 compounds were obtained in which different metabolites such as monoterpenes, sesquiterpenes, and different kinds of phthalides such as 3-butylphthalide, 3-butylidenephthalide, *cis*-butylidenephthalide, and (*Z*)-ligustilide were determined. Consequently, (*Z*)-undec-6-en-2-one (15.08%) and (*Z*)-butylidenephthalide (4.348%) were the main compounds. (*Z*)-undec-6-en-2-one has not been detected before in plants from Apiaceae family [17]. 

**Figure 1:**
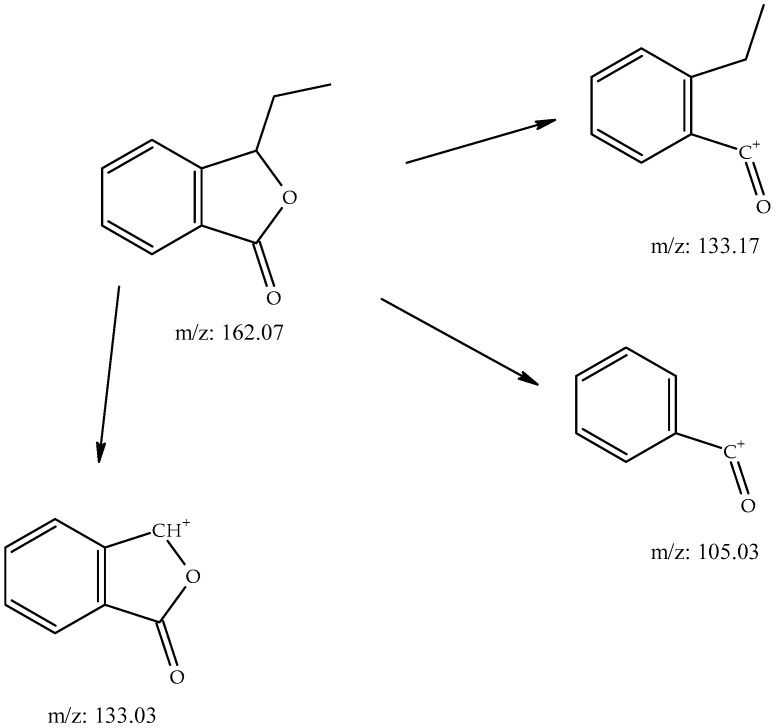
Proposed EI-MS fragmentation of ethylphthalide on the basis of https://doi.org/10.1124/dmd.112.049684, accessed on 1 July 2023.

Hashemi et al. [18] also used SPME in aerial parts of *Kelussia*; however, the results of the present research included most of the compounds. Hashemi et al. [18] established butylphthalide as the most abundant in compound in the leaves, while in the present study, (*Z*)-undec-6-en-2-one was the most frequent compound. Furthermore, in our study, different types of phthalides were determined. Analysis of the spectra obtained by the SPME technique also revealed the presence of ethylphthalide (0.06%), hitherto unpresented in the NIST20 database. It is probably synthesized, such us other phthalides, by condensation of malonyl Co-A via 5-methylorsellinic acid [19]. Its structure was established on the basis of its EI-MS spectrum (Figure 1) according to the fragmentation of phthalidephthalides proposed by Diao et al. [20]. Appropriate cation-radicals with *m*/*z* 162, 133, and finally 105 are in the figure with a proposed structure.

**Figure 2:**
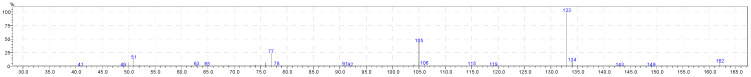
EI-MS of unknown compound (Table 1, SPME) 15.30 min.

**Figure 3:**
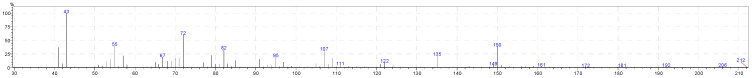
EI-MS of unknown compound (Table 1, SPME) 20.77 min.

**Figure 4:**
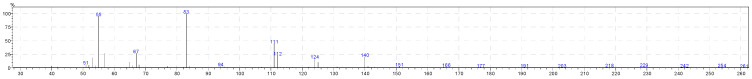
EI-MS of unknown compound (Table 1, SPME) 24.65 min.

### 2.3. Essential Oils Composition

The results obtained by GC-MS (liquid injection) were different in comparison to the SPME technique. This is due to the fact that some EOs are covered by glandular tissues [8]. In some families like Lamiaceae, the essential oils are mostly accumulated in secretory hairs, while in some other families like Apiaceae, the oils are mostly found in secretory sacs or sometimes canals. In Apiaceae leaves, the canals are mostly distributed near the main vein and occasionally in the mesophyll [21]. So, in *Kelussia*, an Apiaceae plant, the analyses based on the oil might lead to the determination of some compounds in secretory sacs or canals. However, in this research, the high quality and accuracy of the SPME-arrow technique provided a relatively a high number of new compounds. 

Based on the GC-MS analysis of essential oils, (*Z*)-ligustilide was determined to be the major compound in the leaves (Table 5). The results are in the range (51.3–58.7%) of those reported in previous studies [3,10]. However, in the present research, new compounds were also determined that were not previously reported. The new components were 3-ethylisobenzofuran-1(3H)-one, (E)-ligustilide, and (E)-n-butylidene phthalide. Asuming et al. [22] also reported different types of phthalides in four *Lomatium* species from North America. Many alkenyl or alkyl phthalide derivatives possess strong antibacterial, antifungal, and cytotoxic activity. What makes these derivatives unique is that some monomeric phthalides have a positive impact on the central nervous system and possess a proven effect in slowing down Parkinson and Alzheimer disease [11].

**Figure 5:**
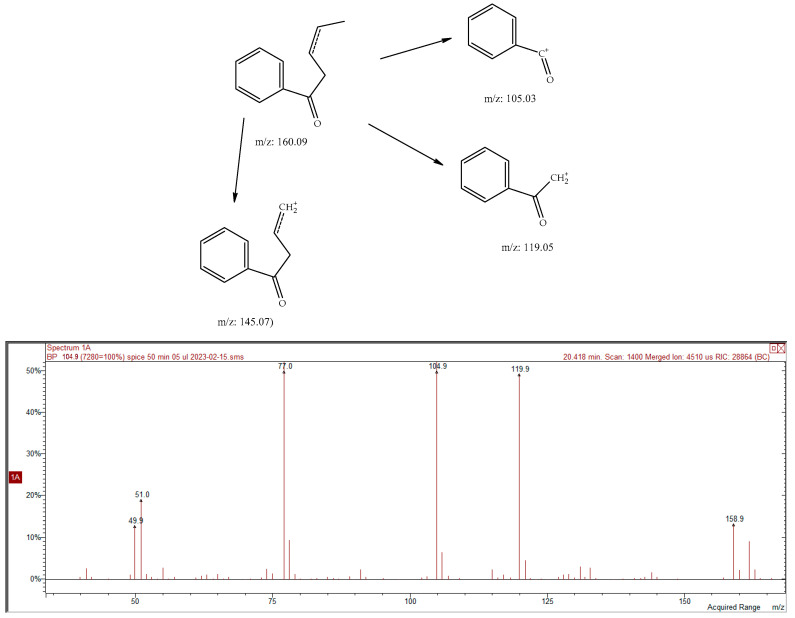
Proposed fragmentation of compound 20.44 min, 3-penten-1-one, 1-phenyl- and EI-MS spectrum of compound.

**Figure 6:**
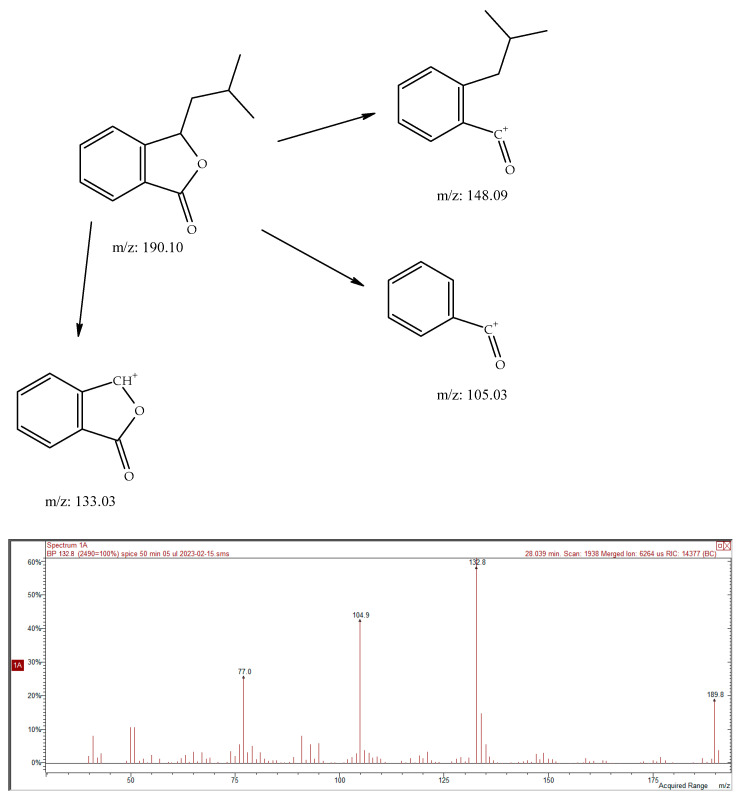
Proposed fragmentation of compound 28.04 min, isobuthylphthalide and EI-MS spectrum of compound.

The presence of phthalide compounds in *Kelussia* was previously proven by Pan et al. [23]. They also reported the hepatotoxicity of furan-containing components in the cortex Dictamni and the correlation with metabolic activation. The new reports also highlighted the importance of ligustillides as an antidepressant compound using bioinformatic methods to determine the mechanisms [24]. Furthermore, anti-inflammatory and antioxidant activities have also been reported for these compounds [25]. In addition, two never before described phthalide derivatives (ethyl and butenylphthalides, 0.06 and 0.09%, respectively) were found in *Kelussia* EO. Fragmentation of the mass of these compounds with RT 28.02 min revealed the characteristic butylphthalide pattern *m*/*z*: 190/148/133/105. Due to the high similarity (in comparison of the EI-MS spectrum presented in NIST20 *n*-buthylphthalide and the lower (about 50) retention index value), we assume the isobutyl isomer to be the only one possible. Also, the characteristic spectra (*m*/*z*: 160/145/119/119) with a pattern similar to valerophenone could suggest the presence of its unsaturated derivative. The proposed EI-MS fragmentation of ethylphthalide is illustrated in Figure 1. Furthermore, the EI-MS of unknown compounds (Table 1, SPME) at 20.77 min and (Table 1, SPME) 24.65 min are shown in Figure 2, Figure 3 and Figure 4, respectively. Finally, the proposed fragmentation of some compounds is also illustrated in Figure 5 and Figure 6.

## 3. Materials and Methods

### 3.1. Liquid Chromatography Mass Spectrometry 

#### 3.1.1. Sample Preparation 

For this purpose, 1 g of *Kelussia* dried leaves was used for the extraction. Consequently, 15 mL of pure methanol (Sigma-Aldrich, Steinheim, Germany) was added to the ground samples. After shaking for 1 h on the shaker (120 rpm), the extracts were separated from the residues. This part was repeated three times. Then, rotary evaporator (Heidolph, Germany) was used to dry the extract. Finally, the dried samples were resolved in pure methanol. Then, after centrifuge (12,000 rpm, 5 min), the pure extract was diluted 1:10 and was used for the LC-MS injection. 

#### 3.1.2. Instrumental Analysis

The analysis of phenolic acids and flavonoids content was carried out with the LC-MS 8045 apparatus (Shimadzu, Kyoto, Japan) equipped with ESI type ion source. The separation of analytes was performed with a Prominence-I LC-2030C 3D Plus (Shimadzu, Kyoto, Japan) unit equipped with a Kinetex 2.6 µm C18 100A 100 × 3.0 mm column with a Security Guard ULTRA 3 mm (Phenomenex, Torrance, CA, USA).

We used 0.1% aqueous formic acid (A) and methanol with 0.1% of formic acid (B) (Sigma-Aldrich, Steinheim, Germany) as mobile phases. The gradient programme was as follows: from 10% to 20% B in 0–5 min; from 20% to 60% B in 5–10 min; from 60% to 10% B in 10–13 min; 10% B up to 17 min. The mobile phase flow was 0.35 mL·min^−1^ at 35 °C.

The screening of non-volatiles was carried out with polyphenols: standard mixture of phenolic acids and alcohols and polyphenols: standard flavonoids mixtures (MetaSci, Toronto, ON, Canada).

The identified compounds were quantified using the MRM mode (Table 1) referring to the calibration curve. The analyses were performed in three repetitions.

### 3.2. HS-SPME Arrow GC/MS Analysis

#### Sample Preparation

First, 50 mg of dried sample (not ground) were used for the SPME analysis. The samples were placed in special glasses for SPME along with the internal standard (2-undecaneone). Prior to the analysis, the lack of internal standard was proven. 

Volatile extraction was carried out with 1.10 mm DVB/C-WR/PDMS SPME Arrow fibre (Shimadzu, Kyoto, Japan). Before the analysis, the samples were preconditioned at 45 °C and then the volatiles were extracted for 30 min at the same temperature. After the extraction, the analytes were desorbed under GC/MS injector conditions.

Volatile analysis was performed with Shimadzu GCMS QP 2020 Plus (Shimadzu, Kyoto, Japan) equipped with a Zebron ZB-5 MSi capillary chamber (30 m × 0.25 mm × 0.25 μm; Phenomenex, Torrance, CA, USA). The injection was carried out at 250 °C and split 40; helium with a column flow of 1.0 nL·min^−1^ was used as a carrier gas. The analytes separation was performed with the following temperature program: 50 °C, then to 130 °C at a rate of 4 °C·min^−1^, then to 180 °C at a rate of 10 °C·min^−1^, then to 280 °C at a rate of 20 °C·min^−1^. The MS operational conditions were as follows: interface temperature 250 °C; ion source temperature 250 °C; scan mode 40–400 *m*/*z*.

The identification of analytes was performed by comparison of the experimentally obtained mass spectra and linear retention indices (±10) with those available in the library of Flavours and Fragrances of Natural and Synthetic Compounds 3.0 (FFNSC 3.0) and the NIST/EPA/NIH EI Mass Spectral Library (NIST 20), Gaithersburg, MD, USA.

The quantification of volatiles was based on GC-MS signals. The quantification was carried out with the peak area normalization method calculated against the peak area of the internal standard. The analyses were performed in three repetitions. 

Authentic standards of volatiles were bought from MetaSci (Toronto, ON, Canada), Aldrich and UQF (Wroclaw, Poland). 

### 3.3. Essential Oil Isolation

The distillation was carried out on *Kelussia* leaves using a Deryng-type apparatus based on the method described by Pachura et al. [8]. Accordingly, 100 g of dried *Kelussia* leaves, along with 1 mg of undecan-2-one (Sigma-Aldrich, Steinheim, Germany) as an internal standard, were applied. The distillation was carried out in a round bottom flask containing 400 mL of distilled water. Hydro-distillation was carried out for 1.5 h after reaching the boiling point and then the essential oils were transferred to a container and stored at −18 °C. The distillations were run in triplicate.

### 3.4. GC-MS Analysis

Essential oils were analyzed using a gas chromatograph coupled to a mass spectrometer (Shimadzu GC-MS QP 2020, Shimadzu, Kyoto, Japan). Compounds separation was carried out using a Zebron ZB-5 MSi capillary column (30 m × 0.25 mm × 0.25 µm; Phenomenex, Torrance, CA, USA). GC-MS analysis parameters were as follows: scan range 35–320 *m*/*z* in mode of 3 scans·s^−1^. Helium was used as the carrier gas at a flow rate of 1.01 mL·min^−1^, with a split ratio of 1:30. The temperature for GC was as follows: from 45 °C as initial temperature to 150 °C at a rate of 2 °C·min^−1^, then to 270 °C at a rate of 15 °C for 5 min. The injection volume was 1 µL. The mass unit was set at an ion source temperature of 240 °C and an ionization voltage of 70 eV. The oil retention indices were calculated using the retention time of the *n*-alkane series (C7–C24) (Adams, 2007) as well as NIST as was described in section HS-SPME Arrow analyses. The amount of oils was obtained from the GC/MS peak using Spectrus software. 

## 4. Conclusions

In the present investigation, comprehensive research was carried out to assess most of the volatile and non-volatile compounds in *Kelussia odoratissima*, an endangered endemic species of Iran. Accordingly, for the first time, the phenolic and flavonoid compounds were determined based on the LC-MS analysis in which chlorogenic acid, neohesperidin, and diosmin were the most abundant compounds for phenolics and flavonoids. Furthermore, based on a thorough analysis of SPME, 78 compounds that belonged to different groups of terpenes and phthalides were determined. Furthermore, the GC-MS analysis also supported the SPME data in most cases. Finally, the introduction of new volatiles and non-volatiles in this valuable endemic plant can provide an insight for further research into pharmaceutical and food science in the future.

## Figures and Tables

**Table 1:** Details of the MRM mode analysis for identified compounds.

Compound	Precursor *m*/*z* [M−H]^−^	Product *m*/*z*	Relative Product Ions Abundance [%]	Q1 Pre Bias ^1^ (V)	CE ^2^	Q3 Pre Bias ^3^ (V)
3-Hydroxyflavone *	238.9	164.9	100	−12.0	−33.0	−25.0
		120.95	51	−26.0	−29.0	−18.0
		104.95	25	−12.0	−30.0	−15.0
Flavone *	222.9	76.8	100	−11.0	−38.0	−29.0
		120.95	7	−11.0	−29.0	−18.0
		64.8	9	−11.0	−46.0	−24.0
Quercetin	300.9	150.9	100	14.0	24.0	28.0
		178.95	48	14.0	17.0	11.0
		106.9	25	14.0	29.0	20.0
Neohespederin dihydrohalcone	611.1	303.0	100	28.0	35.0	20.0
		125.0	27	28.0	45.0	24.0
		165.9	5	28.0	55.0	16.0
Rutin	609.0	301.0	57	30.0	27.0	19.0
		285.9	6	30.0	44.0	17.0
		299.05	100	28.0	28.0	19.0
Neohesperidin	609.0	300.95	100	28.0	27.0	20.0
		286.0	30	30.0	43.0	17.0
		164.0	11	28.0	54.0	16.0
Polydatin	389.0	321.0	50	19.0	12.0	23.0
		343.1	100	20.0	14.0	24.0
Diosmin	607.0	299.2	100	30.0	30.0	20.0
		283.7	29	30.0	49.0	18.0
*p*-Coumaric acid	163.0	119.05	100	15.0	16.0	22.0
		92.95	7	16.0	31.0	17.0
		91.05	14	15.0	29.0	13.0
Ferullic acid	193.4	134.0	100	12.0	14.0	26.0
		177.95	61	12.0	15.0	30.0
		149.05	19	12.0	13.0	14.0
Caffeic acid	179.5	134.95	100	11.0	16.0	25.0
		134.65	27	11.0	30.0	23.0
		106.95	3	11.0	22.0	19.0
Chlorogenic acid	353.0	191.0	100	16	17.0	20.0
		84.95	21	16	43.0	16.0
		92.95	5	16	46.0	17.0
Acetylphloroglucinol	167.5	123.0	100	11.0	17.0	24.0
		80.95	29	10.0	22.0	15.0
		83.0	31	11.0	24.0	16.0
*m*-Coumaric acid	163.4	119.0	100	10.0	15.0	23.0
4-Methylsiringol	167.3	123.0	100	26.0	13.0	26.0
		108.0	64	10.0	15.0	11.0

* Compounds analysed with a positive ionization mode [M + H]^+^; ^1^ voltage promotes the ionization of the precursor ion; ^2^ collision energy; ^3^ voltage promotes the ionization of the product ion.

**Table 2:** The amount of phenolic acids in the studied *Kelussia* leaves.

Compounds	RT [min]	Concentration (µg/gDw)
Chlorogenic acid	4.65	303.08 ± 1.9
Vanillic acid	4.97	28.02 ± 0.9
4-Methylsiringol	4.97	57.03 ± 0.85
Acetylphloroglucinol	4.98	21.66 ± 0.64
Caffeic acid	5.20	1.94 ± 0.44
*p*-Coumaric acid	6.42	2.14 ± 0.53
*m*-Coumaric acid	6.42	2.93 ± 0.25
Ferullic acid	6.84	62.60 ± 1.2

**Table 3:** The flavonoids amount in the studied *Kelussia* leaves.

Compounds	RT [min]	Concentration (µg/gDw)
Polydatin	1.40	1.15 ± 0.34
Neohesperidin dihydrochalcone	6.46	0.09 ± 0.12
Neohesperidin	6.46	38.37 ± 0.87
Rutin	6.48	23.08 ± 0.11
Flavone	8.76	1.07 ± 0.86
3-Hydroxyflavone	8.85	0.20 ± 0.09
Diosmin	9.84	28.62 ± 0.76
Quercetin	9.88	0.87 ± 0.08

**Table 4:** SPME profile of the *Kelussia* leaves.

Nr	Peak Name	tR (min)	KI Exp.	KI Lit.	Area (%) ^e^	Identification	Similarity ^d^
1	Isobutyric acid	3.19	770	756	1.22	S, KI, MS	95
2	Butanoic acid	3.63	801	802	13.75	S, KI, MS	92
3	*n*-Hexanal	3.95	803	801	2.10	S, KI, MS	90
4	Isovaleric acid	4.61	845	850	1.96	S, KI, MS	92
6	Butanoic acid, 2-methyl-	4.87	855	861	2.26	S, KI, MS	89
7	2-(*E*)-Hexenal	5.08	862	864	0.01	S, KI, MS	86
8	Pentanoic acid	5.55	899	901	1.17	S, KI, MS	84
9	trans-2-Pentenoic acid	5.83	905	909	1.37	S, KI, MS	85
11	Hexanal, 3-methyl-	6.17	908	910	0.72	S, KI, MS	90
12	Acetylfuran	6.47	926	911	0.26	KI, MS	89
13	Benzene, propyl-	6.57	926	953	0.21	KI, MS	91
14	2-Heptenal, (*E*)-	7.75	960	958	0.16	KI, MS	93
15	Benzaldehyde	7.92	970	962	0.23	S, KI, MS	95
16	1-Heptanol	8.12	976	970	0.18	S, KI, MS	90
17	4-Octanone	8.24	979	975	0.61	KI, MS	92
18	Hexanoic acid	8.34	982	990	1.41	S, KI, MS	87
19	5-Hepten-2-one, 6-methyl-	8.67	991	986	0.23	KI, MS	96
20	Furan, 2-pentyl-	8.81	997	993	0.12	KI, MS	91
21	2-Ethylhexenal	9.11	1004	999	0.062	KI, MS	93
22	Octanal	9.17	1006	1003	1.96	S, KI, MS	94
23	3-Hexenoic acid, (*E*)-	9.42	1014	1021	0.70	KI, MS	91
24	*p*-Cymene	9.90	1031	1025	0.09	S, KI, MS	94
25	Limonene	10.03	1035	1031	0.21	S, KI, MS	90
26	Benzyl alcohol	10.23	1041	1036	0.31	S, KI, MS	93
28	Phenylethanal	10.59	1052	1045	0.68	KI, MS	89
29	4-Hexanolide	10.95	1062	1057	0.06	KI, MS	87
30	2-Octenal, (*E*)-	11.02	1062	1060	0.16	S, KI, MS	91
31	2-Acetylpyrrole	11.105	1068	1663	0.41	KI, MS	88
32	Octanol	11.44	1075	1071	0.48	S, KI, MS	90
33	3,5-Octadien-2-one	12.24	1097	1091	0.57	KI, MS	91
34	Linalool	12.44	1099	1099	0.08	S, KI, MS	95
35	Nonanal	12.59	1099	1104	4.95	S, KI, MS	94
36	Octadienol <(2*E*,4*E*)->	12.79	1114	1116	0.28	S, KI, MS	92
37	Phenylethyl Alcohol	12.96	1119	1116	0.67	S, KI, MS	90
38	Camphor	14.09	1150	1142	0.18	S, KI, MS	94
39	Benzene, pentyl-	14.46	1163	1157	0.55	KI, MS	90
40	Octanoic acid	14.85	1174	1180	0.68	S, KI, MS	89
41	Menthol	15.02	1178	1174	0.38	S, KI, MS	91
42	Unknown ^a^	15.30	1186	n.d.	0.18	-	-
43	*p*-Cymen-8-ol	15.47	1190	1183	0.18	S, KI, MS	93
44	Estragole	15.94	1203	1196	0.10	S, KI, MS	91
45	Decanal	16.13	1207	1206	0.38	S, KI, MS	89
46	Carvone	17.52	1253	1246	1.19	S, KI, MS	92
47	Piperitone oxide	17.91	1263	1256	0.70	S, KI, MS	93
48	2-Decenal, (*E*)-	18.06	1271	1263	0.49	KI, MS	92
49	*n*-Nonanoic acid	18.20	1271	1273	0.67	S, KI, MS	88
50	(*Z*)-6-Undecen-2-one	18.59	1282	1274	15.09	KI, MS	92
51	1-Tridecyne	18.70	1286	1297	0.55	KI, MS	90
52	IS	19.13	1297	1294	15.41	S, KI, MS	95
53	unknown ^b^	20.77	1352	n.d.	0.26	-	-
54	Ylangene	20.98	1365	1372	0.25	KI, MS	92
55	Valerophenone	21.15	1368	1373	2.17	KI, MS	91
56	2-Undecenal, (*Z*)-	21.30	1373	n.d.	0.60	KI, MS	93
57	Copaene	21.69	1387	1376	1.28	KI, MS	90
58	Hexanoic acid, hexyl ester	21.85	1391	1384	0.13	KI, MS	89
59	Tetradecane	22.14	1400	1400	0.26	S, KI, MS	85
60	Dodecanal	22.38	1411	1408	0.30	S, KI, MS	92
61	α-Barbatene	22.73	1435	1435	0.27	KI, MS	90
62	Ethylphthalide *	23.38	1464	n.d.	1.26	MS	-
63	Acoradien	23.66	1480	1471	0.87	KI, MS	93
64	4-epi-α-Acoradiene	23.81	1486	1475	0.81	KI, MS	91
65	α-Curcumene	23.90	1491	1483	0.59	S, KI, MS	95
66	Cuparene	24.39	1521	1505	2.19	KI, MS	93
67	Cubebol	24.54	1532	1515	0.36	KI, MS	89
68	unknown ^c^	24.65	1538	n.d.	1.20	-	-
69	Kessane	24.78	1548	1537	2.24	KI, MS	90
70	Actinidiolide, dihydro-	24.84	1551	1534	1.04	KI, MS	93

IS: internal standard; unknown ^a–c^ spectra are illustrated in Figure 2, Figure 3 and Figure 4; S: authentical standard, KI: Kovat’s index, MS: mass spectrum; ^d^ similarity match according to LabSolution v. 4.45; ^e^ according to TIC-MS chromatogram; * new identified compound.

**Table 5:** The essential oil composition of *Kelussia* based on GC-MS on distilled essential oil.

Peak Name	tR (min)	KI Exp.	KI Lit.	Area (%)	Identification	Similarity ^a^
Isocumene *	7.217	956	953	0.087	KI, MS	94
Heptenol (3-*Z*)	7.286	959	954	0.039	KI, MS	92
4-Octanone	7.734	974	976	0.042	KI, MS	95
2-Ethyl-2-hexenal	8.576	1001	990	0.097	KI, MS	91
Limonene	9.491	1033	1031	0.073	S, KI, MS	95
Terpinolene	11.45	1090	1088	0.068	S, KI, MS	97
5-Pentylcyclohexa-1,3-diene	13.802	1162	1161	0.854	KI, MS	91
Carvone	16.729	1248	1242	0.09	S, KI, MS	93
(*Z*)-Undec-6-en-2-one	17.802	1279	1279	2.233	KI, MS	90
2-Undecanone (IS)	18.381	1295	1294	7.489	S, KI, MS	93
2-Methoxy-4-vinylphenol	19.116	1318	1318	0.508	KI, MS	89
3-Penten-1-one, 1-phenyl- *	20.433	1360	n.d.	0.383	MS	92
Copaene	21.108	1380	1376	0.209	KI, MS	94
*trans*-β-Caryophyllene	22.504	1424	1419	0.069	S, KI, MS	94
β-Barbatene	23.245	1449	1451	0.062	KI, MS	92
Ethylphthalide *	23.459	1455	n.d.	0.064	MS	92
Acoradien	23.984	1472	1471	0.134	KI, MS	-
α-Curcumene	24.405	1485	1483	0.085	S, KI, MS	91
β-Ionone	24.816	1498	1491	0.12	S, KI, MS	96
Cuparene	25.14	1509	1505	0.329	KI, MS	93
Cubebol	25.478	1521	1515	0.119	KI, MS	91
δ-Cadinene	25.644	1527	1524	0.234	KI, MS	89
Kessane	25.8	1533	1537	0.587	KI, MS	92
Caryophyllene oxide	27.42	1587	1581	0.181	S, KI, MS	92
Isobutylphthalide	28.025	1608	n.d.	0.098	MS	-
Humulene epoxide II	28.164	1613	1608	0.127	KI, MS	94
γ-Eudesmol	28.489	1626	1630	0.042	KI, MS	92
Cubenol	28.686	1633	1642	0.077	KI, MS	88
Gossonorol	28.926	1642	1640	0.085	KI, MS	92
3-*n*-Butylphthalide	29.337	1656	1656	0.682	S, KI, MS	93
(*Z*)-*n*-Butylidene phthalide	29.97	1678	1675	11.57	S, KI, MS	95
unknown	30.096	1683	n.d.	0.463	-	-
(*E*)-*n*-Butylidene phthalide	31.16	1723	1722	2.092	KI, MS	93
(*Z*)-Ligustilide	31.797	1748	1740	50.479	S, KI, MS	92
(*E*)-Ligustilide	33.195	1802	1810	1.455	S, KI, MS	93

S: authentical standard, KI: Kovat’s index, MS: mass spectrum; ^a^ similarity match according to LabSolution v. 4.45. * The new identified compounds.

## Data Availability

The data are available upon request.
